# A variant by any name: quantifying annotation discordance across tools and clinical databases

**DOI:** 10.1186/s13073-016-0396-7

**Published:** 2017-01-26

**Authors:** Jennifer L. Yen, Sarah Garcia, Aldrin Montana, Jason Harris, Stephen Chervitz, Massimo Morra, John West, Richard Chen, Deanna M. Church

**Affiliations:** 1 Personalis, 1330 O’Brien Drive, Menlo, Park, CA 94025 USA; 2 10X Genomics, 7068 Koll Center Pkwy #401, Pleasanton, CA 94566 USA

**Keywords:** HGVS, Clinical testing, Genomics, Annotation, Sequencing, Syntax, Precision medicine, Variant

## Abstract

**Background:**

Clinical genomic testing is dependent on the robust identification and reporting of variant-level information in relation to disease. With the shift to high-throughput sequencing, a major challenge for clinical diagnostics is the cross-identification of variants called on their genomic position to resources that rely on transcript- or protein-based descriptions.

**Methods:**

We evaluated the accuracy of three tools (SnpEff, Variant Effect Predictor, and Variation Reporter) that generate transcript and protein-based variant nomenclature from genomic coordinates according to guidelines by the Human Genome Variation Society (HGVS). Our evaluation was based on transcript-controlled comparisons to a manually curated set of 126 test variants of various types drawn from data sources, each with HGVS-compliant transcript and protein descriptors. We further evaluated the concordance between annotations generated by Snpeff and Variant Effect Predictor and those in major germline and cancer databases: ClinVar and COSMIC, respectively.

**Results:**

We find that there is substantial discordance between the annotation tools and databases in the description of insertions and/or deletions. Using our ground truth set of variants, constructed specifically to identify challenging events, accuracy was between 80 and 90% for coding and 50 and 70% for protein changes for 114 to 126 variants. Exact concordance for SNV syntax was over 99.5% between ClinVar and Variant Effect Predictor and SnpEff, but less than 90% for non-SNV variants. For COSMIC, exact concordance for coding and protein SNVs was between 65 and 88% and less than 15% for insertions. Across the tools and datasets, there was a wide range of different but equivalent expressions describing protein variants.

**Conclusions:**

Our results reveal significant inconsistency in variant representation across tools and databases. While some of these syntax differences may be clear to a clinician, they can confound variant matching, an important step in variant classification. These results highlight the urgent need for the adoption and adherence to uniform standards in variant annotation, with consistent reporting on the genomic reference, to enable accurate and efficient data-driven clinical care.

**Electronic supplementary material:**

The online version of this article (doi:10.1186/s13073-016-0396-7) contains supplementary material, which is available to authorized users.

## Background

High-throughput sequencing has transformed the landscape of clinical genetic testing. This strategy, combined with the completion of massive public profiling datasets (ExAc [1], 1000 Genomes [2]), has dramatically changed our approach towards cancer treatment and the diagnosis of inherited disease. A major challenge in the analysis of this throughput and volume of data is integrating variant level information from the wealth of clinical and biological insight accumulated over decades of research, particularly those from recent, large sequencing studies. Describing a variant’s location is a fundamental part of a clinical assessment, yet the practice remains inconsistent and continues to evolve.

Specifically, the clinical genomics community faces an enormous hurdle, which is integrating data generated prior to the availability of a robust human reference assembly with that generated using modern methods. Standards and guidelines for describing variants at the genomic, transcript (coding), and protein levels are provided by the Human Genome Variation Society (HGVS) [3], which developed and published initial recommendations in 1998–2000, when testing was still largely transcript- rather than genome-based [3, 4]. As laboratories shifted to high-throughput sequencing, variant analysis transitioned to the genome level, confounding comparisons with reports generated from previous transcript-based assays. A recent update to the initial 2000 guidelines was published last year to reflect changes in nomenclature descriptions, including eliminating the use of “IVS” to indicate intronic sites (e.g., IVS4-2A > C), and the use of “X” to reference the termination codon. Further, during the submission and review of this manuscript, a new website was launched with a more streamlined description of compliant syntax (http://varnomen.hgvs.org).

**Fig. 1:**
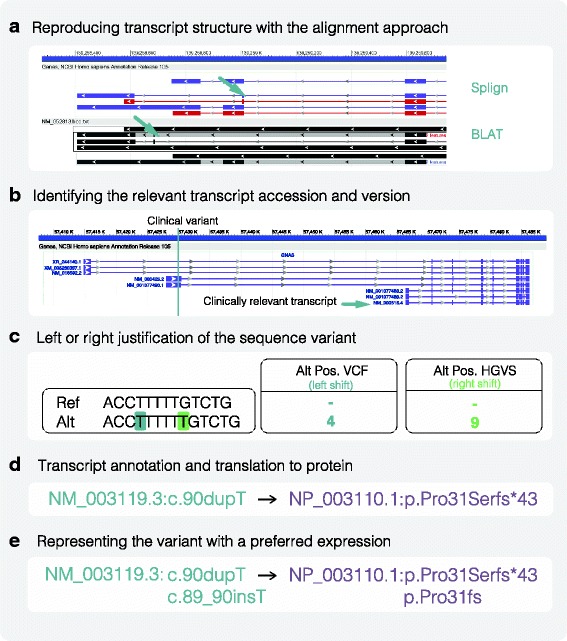
Factors affecting HGVS syntax generation. **a** Transcript alignment approach can impact the transcript exon structure. Alignment of cDNA sequence by Splign and BLAT to the genome results in a 10-kb difference in an exon positioning in the CARD9 gene (*green arrow*). **b** Transcript accession can impact the variant association and HGVS syntax. Here, the identified GNAS variant is outside the clinically relevant transcript. Small changes in versions may also impact the coding sequence. **c** In the context of nucleotide repeats, variant justification can affect the variant’s position. **d** Transcript annotation directly impacts its translation to a protein expression. Incorrect transcript annotation can lead to incorrect protein syntax. **e** Representing the variant in a particular expression. There are different ways of expressing the same coding or protein variant

Reconciling variant coordinates from the transcript to the genome, and vice versa, is not an unambiguous task. Requisite information about the genomic and transcript sequence accessions, their versions, and the alignments to relate the two sequences are not always reported in publications (Fig. 1a, b). Alignment of cDNA to the genome remains challenging and can result in substantially different exon structures depending on the alignment tool used (Fig. 1a) [5, 6]. In addition, variant reporting standards for the Variant Call Format (VCF), a format designed to store genomic variation, are different from those for HGVS, a format that was developed by clinical laboratories to describe transcript and protein variants. In the context of nucleotide repeats, VCF shifts left with respect to the genome, while HGVS shifts right with respect to the gene or transcript, meaning secondary information is required to calculate the HGVS expression (Fig. 1c). Variants can therefore have completely different locations depending on their accession, version, and alignment.

Even in relation to the same transcript, a variant can have multiple representations. HGVS expressions can have long and short forms, preferred and non-preferred syntax, and describe amino acids by their triple (e.g., Glu) or a single letter designation (e.g., E) (Fig. 1d, e). In a survey by Deans et al. (2016) [7], 20 laboratories reported the HGVS syntax for a single variant in 14 different ways. An evaluation of over 140 molecular pathology laboratories in Europe and the UK revealed substantial errors in the reporting of HGVS variant descriptions for the EGFR gene [8]. While a subset of the syntax differences may be interpretable to a clinician (e.g., p.R154X and p.ARG154*), the majority are not interpretable and confound searches used to determine if a variant has been seen before. Even a single character change can confound a search if that variant is stored using a different form, even when both forms conform to the HGVS recommendations.

We currently have many tools for producing HGVS syntax, including SnpEff [9], Variant Effect Predictor (VEP) [10], Annovar [11], Variation Reporter (VR) [12], Mutalyzer [13], and packages developed by individual clinical laboratories such as Invitae [6] and Counsyl [14]. While the performance of different genomic variant callers have been well-studied [15, 16], the accuracy and consistency of HGVS generation tools remain unknown.

Previous comparison of Annovar and VEP revealed substantial differences in annotation based on choice of transcript [17]. This low concordance, combined with the increasing demand for automated syntax generation, prompted our re-evaluation of the performance of well-supported, open source tools. We considered only freely available tools as they would have the largest reach. Additionally, we wished to focus on annotation differences that can occur even when the same transcript is used and any impact on protein consequence annotations. In this study, we compare the concordance of variant nomenclature generated by VEP [10], SnpEff [9], and VR, benchmarked by a curated “truth” set and variant annotations described in large public datasets for germline (ClinVar) and cancer (COSMIC) variant descriptions. We find that while the tools SnpEff and VEP produce comparable results, significant discordance remains in variant annotation among the tools, public resources, and literature.

## Methods

### Datasets

We curated a test set of 126 variants to establish a ground truth set with which we can evaluate the accuracy of the tools (Additional file 1: Table S1; Additional file 2). Fifty variants were selected from public repositories: ClinVar [18], dbSNP [19], COSMIC [20], My Cancer Genome [21], Emory Genetics Laboratory (EmVClass) database [22], and Leiden Open Variation Database (LOVD) [23] (Additional file 1: Table S1). We added 76 synthetic variants to ensure representation across variant types and genomic features. Genomic, coding, and protein nomenclature for all variants were generated using a combination of the Mutalyzer webservice [24] and Variation Viewer [25]. Effect impact was determined based on the protein syntax and sequence ontology (SO) [26].

We used the ClinVar GRCh37 VCF and annotations from the tab separated file downloaded from the FTP site [27] (5th January 2016 release). We used the rsid and alternative allele to connect variants between the two files. (For deletion variants, a hyphen (“-”) was used to represent the alternative allele.) We downloaded the COSMIC GRCh37 VCF and CosmicCompleteExport.tsv file from the COSMIC website [28] (v75) and connected variants using the COSMID.

### VCF normalization

We used vt-normalize [29] to left-justify all variants in each of the dataset VCFs used as input for annotation. A breakdown of insertions and deletions (indels) for each dataset and the number normalized are represented in Additional file 1: Table S2.

### Tools used

We ran SnpEff (v4.1 L) [9, 30], VEP (v82) [10], and VR [12] on our ground truth set, and subsequently only SnpEff and VEP on the ClinVar and COSMIC datasets. The Snpeff database was built using the NCBI GRCh37 GFF corresponding to the NCBI annotation “*Homo sapiens* 105”. The SnpEff database for Ensembl transcripts was built using the GRCh37 Ensembl transcript GFF (v82) [31]. Further details about our SnpEff installation are described in Additional file 3.

We ran VEP with the corresponding RefSeq or Ensembl cache (v83). For all tools we used NCBI GRCh37p13 as the input reference genome. We ran VEP with the additional options: --hgvs, --vcf, --allele_number, and --variant_class. We ran SnpEff using the default command line.

### Assessment of syntax

**Fig. 2:**
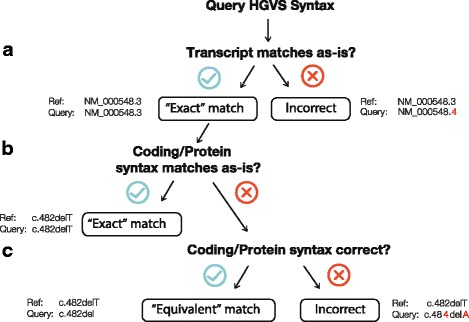
Methodology of HGVS syntax comparison. To compare two HGVS expressions in our dataset, we applied the following assessments. **a** The query transcript must match the reference transcript. If the accession or version does not match, the variant is not assessed. **b** If the syntax for both expressions correspond as-is, the match is “exact”. **c** If the syntax for both expressions are equivalent, the match is “equivalent”. If the syntax is not an alternative expression of the other HGVS variant, the match is “incorrect”

To assess the performance of the variant annotation tools, we performed string match comparisons between the output and the reference syntax with variants described on the same transcript accession and version (Fig. 2). Annotations were evaluated according to the HGVS guidelines [32, 33]. Variant annotations were labeled as “exact” matches when the HGVS string and the query annotation matched as-is. If the string did not match perfectly, but could be transformed to the query string by applying HGVS recommendations, the tool’s annotation was labeled “equivalent”. For this study, both exact and equivalent annotations are regarded as correct. Although the HGVS recommends that proteins without any experimental evidence should be described in parentheses, we decided to omit the parentheses in syntax comparison as none of the tools or databases compared employed this practice. Also, HGVS states that intronic variants cannot be described with respect to the coding DNA reference sequence because the reference sequence must contain the variant residue described. Because none of the tools or databases used in this study adhere to this guideline, we also omitted this requirement.

## Results

### Comparisons to a ground truth test set

**Fig. 3:**
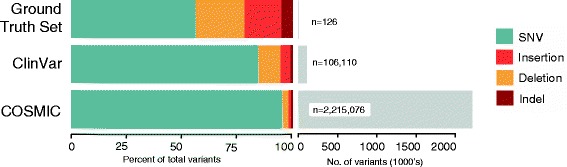
Datasets by composition. Number of variants and distribution of variant types in the Ground Truth, ClinVar, and COSMIC dataset. Note that due to transcript discrepancies, the number of variants evaluated may be less than the number of variants in the input set. *SNV* refers to single nucleotide variant

In order to assess the performance of different variant annotation tools against a ground truth, we used a contrived test set of 126 manually curated variants (Additional file 1: Table S1) comprised of 50 previously reported variants in the literature or databases and an additional 76 synthetic variants targeting a spectrum of variant types (Fig. 3; Additional file 1: Table S3). All annotations were reviewed manually using a combination of the Mutalyzer and Variation Viewer web services. In choosing variants for the ground truth set, we deliberately selected examples covering a variety of edge cases we have encountered in our own laboratory that would be particularly difficult to annotate. The purpose was not only to challenge the limits of the tools, but also for quality assurance of our in-house annotation. Therefore, it must be noted that the composition of the ground truth set, and its evaluation, is not representative of that seen in a typical clinical specimen. However, given high volume and automation, rare instances of edge cases become important in a clinical lab and can be problematic if the annotations are not correctly addressed.

Using the analysis flowchart summarized in Fig. 2, we compared the annotations generated by VR, VEP [10], and SnpEff [9] to the ground truth test set (Additional file 1: Table S4). VEP and SnpEff accept VCF as input files; at the time of analysis, the VR API was limited in its functionality in processing large VCF files. Genomic HGVS expressions were also required as input for Mutalyzer, but we did not assess this tool because it was used, in part, to construct the ground truth set. Further, we did not evaluate SeattleSeq as the documentation does not claim to provide HGVS nomenclature, a requirement for clinical labs. We compared only annotations made on the same RefSeq transcript version.

**Fig. 4:**
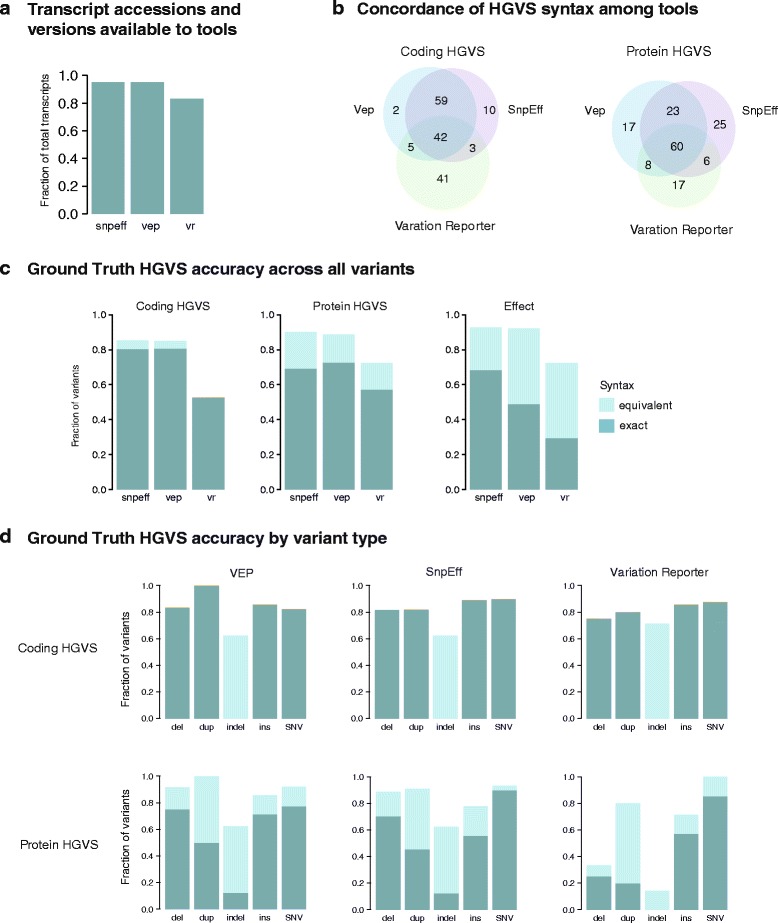
Summary of Ground Truth set HGVS syntax assessment. **a** Fraction of unique transcript accessions and versions in the Ground Truth set that were available to the tools SnpEff (*snpeff*), VEP (*vep*), and Variation Reporter (*vr*). If a transcript was not accessible to the tool, the variant could not be annotated with respect to that transcript. **b** Exact concordance of HGVS syntax at the coding (*left*) and protein (*right*) levels among the tools. **c** Accuracy of annotation across variants (total n = 121) described as exact (*turquoise*) and equivalent (*light turquoise*). Fraction shown is with respect to annotations on the relevant transcripts on the test set. **d** Accuracy of annotation by variant type across the tools. Variant types evaluated were: deletions (*del*), insertions and deletions (*indel*), duplications (*dup*), insertions (*ins*), and single nucleotide variants (*SNVs*)

Although the input transcript alignments for SnpEff and VEP were identical, the tools produced a different number of transcripts and annotations (Fig. 4a). For example, we could not extract the relevant transcript for four variants in the SnpEff output and five from the VEP output, in addition to five variants absent from both tools. The importance of transcript collection was more pronounced for VR, which uses its own in-house alignments. As a result, 26 out of 126 of the test variants could not be assessed by VR because NCBI carried only the most up-to-date transcripts. VR also frequently yielded multiple annotations for a single variant and transcript. In these cases, we chose the first variant in the output to evaluate in this test. In total, only 118 out of the 126 variants were annotated on the relevant transcript for any of the three tools.

A major challenge in comparing nomenclature between tools was evaluating the equivalency of the many HGVS expressions for a given variant. Protein variant syntax was considerably more variable than coding variant syntax: between 13 and 24 protein annotations were described with alternative but equivalent nomenclature across the three tools, compared to at most four variants with coding syntax (Fig. 4c, d). Each tool had distinct frameshift and synonymous annotations; frameshift has both long and short form alternatives, while synonymous variants can be described in several ways, e.g., p.(=), p.=, p.Thr258=, p.Thr258Thr (PTV013, Additional file 1: Table S5). Interestingly, although p.(=) was the preferred HGVS syntax at the time of manuscript preparation (http://www.hgvs.org/mutnomen/), as part of a recently accepted HGVS proposal (SVD-WG001), the new preferred syntax is p.Thr258 = (http://varnomen.hgvs.org/), which we had also argued was a more informative representation. Exact concordance in annotation between SnpEff and VEP was higher at the coding level (92.6% or 100/108 variants) than at the protein level (75% or 81/108) (Fig. 4b). Less agreement was observed between VR and either VEP or SnpEff: at most 44 out of 87 variants matched exactly for coding (≤50.6%) and 55 out of 87 variants for protein syntax (≤63.2%).

For variants in our ground truth set, SnpEff and VEP exhibited comparable accuracy and precision. At the coding level, SnpEff and VEP respectively annotated 89.8% (53/59) and 82.2% (51/62) of substitutions correctly, compared to 100% (48/48) of substitutions for VR. Controlling for transcript accession was important in this comparison: omitting a transcript match resulted in accuracy of between 20 and 30% for coding and 20 and 40% for protein syntax across the tools. For deletions and insertions, VR performed poorly largely due to systematic errors in reporting. VR incorrectly described all but two deletions as indels. The remaining two annotations diverged from HGVS guidelines by omitting the “del” designation altogether (e.g., c.2199-1301GA > A) (PTV019, Additional file 1: Table S5). Duplications were also annotated as indels, but with technically equivalent (and redundant) nomenclature (c.1961dupG as c.1960delCinsCG). Such VR errors at the coding level led to inaccurate protein syntax for 23 variants.

Variant annotations at splice site junctions are complex not only because of the many splice products, but also because of their exception to the 3′ right shifting rule. The HGVS states that insertions and deletions should always be shifted to their 3′ and right-most position relative to the accession sequence, except across intron and exon boundaries. Neither of the tools correctly identified this exception for our test variant, which was a single base deletion at chr12:103234294 (PTV021, rs63186960). The variant should be annotated as NM_000277.1:c.1200-1delG and should not be right-shifted to c.1200delG, as annotated by SnpEff and VEP, nor to c.1201delG as by VR, the latter which is completely outside the repeat sequence. It should be noted that the right-shifting exception does not apply to variants shifting away from the intron–exon border: the correct nomenclature for ClinVar variant NM_017739.3:c.1895 + 1_1895 + 4delGTGA (PTV021, rs63186960) is therefore c.1895 + 5_1895 + 8delGTGA, as asserted by both VEP and SnpEff. VR annotated this variant as c.1895 + 9GTGAC >C.

We also tested the ability of the tools to discriminate between the genomic reference and RefSeq transcript sequences, both of which are independently curated by the NCBI [34]. Since RefSeq transcripts typically receive a high level of manual review, conflicts between the RefSeq and genomic sequence usually reveal an error in the latter. For this reason, we included four test instances of RefSeq-Genomic differences in our ground truth set. Strikingly, none of the four test examples of RefSeq-Genomic differences were identified by either VEP or SnpEff (Additional file 1: Table S5) and were erroneously reported as missense or deletion variants. While VR correctly identified two out of four RefSeq-Genomic differences (the remaining two variants were not annotated), it mistakenly called differences for an additional 22 variants, indicating poor precision for recognizing true differences. HGVS expressions should always reflect the base on the relevant genomic or transcript sequence to avoid asserting variants at positions where there is no change.

Both SnpEff and VEP correctly annotated the phased dinucleotide substitutions, which are variants present in consecutive bases, also known as multinucleotide variants (MNVs; Additional file 1: Table S6). Dinucleotide substitutions are highly prevalent in cancers associated with clear mutagen exposures such as melanoma, lung adenoma, and lung squamous cell carcinoma [35]. Similarly, treatment by the chemotherapeutic agents cisplatin and meclorethamine have also been shown to cause dinucleotide substitutions at appreciable rates [35]. VR incorrectly annotated the phased dinucleotide substitutions as frameshift variants (PTV105). Our results show that for MNVs to be annotated correctly by either tool, they must be phased in the VCF as a block substitution; when the variants are represented on separate rows in the VCF these MNVs will be annotated independently instead of as pairs. This resulted in the incorrect annotation of two BRAF variants (PTV106, PTV107) as p.V600E and p.V600M, when the correct MNV annotation is p.V600K. The accurate annotation of MNVs is therefore dependent on the choice of variant caller to invoke phasing of variants in the VCF. For cancers with a high mutation load, prior phasing for dinucleotide pairs will be especially crucial to circumvent potential clinical oversights [36].

To complement the analysis of protein and coding annotations, we also assessed the variant effects predicted by the tools. Predicted effect is commonly used for evaluating pathogenicity during variant interpretation [37]. In instances where a variant could be associated with two functional consequences (for example, as intronic but also at a slice acceptor site), the annotation was considered to be correct if one association was described. Overall, the accuracy of effect prediction correlated highly with that of protein annotation (Additional file 1: Table S4) even if they are calculated independently [9]. Compared to coding and protein syntax, efforts among tools to converge on a standardized set of variant effect annotations were far more evident (Additional file 1: Table S4; Additional file 4: Figure S1).

### Comparison with the ClinVar dataset

Having established a baseline accuracy for automated syntax generation, we sought to assess the syntax concordance of these tools with those in public datasets, which would provide us a wealth of annotation comparisons that are clinically relevant. We started with ClinVar [18], a large public archive of variant and disease relationships that is widely used for evaluating Mendelian disease. Although the database relies heavily on submissions from publications and clinical laboratories, there are on-going efforts to harmonize these variants by curators at the NCBI. Of the 106,110 small variants in the ClinVar VCF, the vast majority are SNVs (84%); the rest comprise a smaller number of deletions (10%), duplications (3.3%), insertions (1%), and indels (1%) (Fig. 3b). We evaluated the performance of VEP and SnpEff on the ClinVar dataset (Additional file 5); because of the limited functionality and long running time of the VR tool (Additional file 1: Table S7), we did not include it in subsequent annotation assessments.

**Fig. 5:**
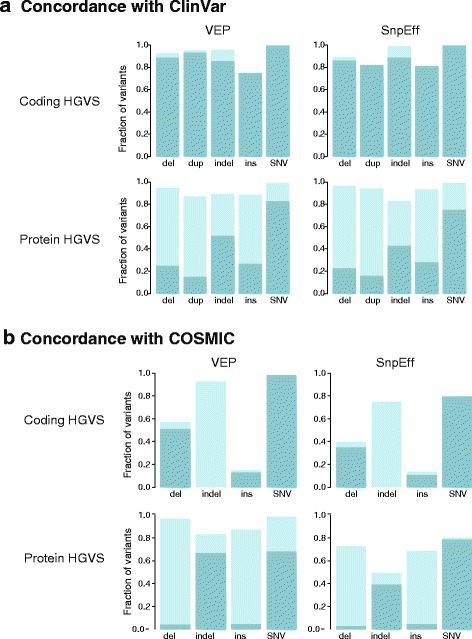
ClinVar and COSMIC HGVS syntax assessment. **a** Overall concordance in syntax by variant type between the tools and ClinVar. **b** Overall concordance in syntax by variant type between tools and COSMIC. All duplications were annotated as insertions in COSMIC. For both ClinVar and COSMIC, coding variants are shown in the *upper panel* and protein variants in the *lower panel*; *bars* represent fraction of exact (*turquoise*) and equivalent (*light turquoise*) matches

Approximately 10% of transcripts in the ClinVar dataset had different versions from those in our input transcript alignment file, which was used to build resources for both VEP and SnpEff (Fig. 5a). Approximately 1.8% of ClinVar transcript accessions were not represented in the alignment input at all. Because of these discrepancies in transcript accession and versions, we could not assess the SnpEff or VEP annotations for 7 and 7.5%, respectively, of ClinVar variants, again underscoring the importance of the input transcript set.

Overall concordance for both SnpEff and VEP was remarkably high, which can be attributed to the dominance of SNVs in the dataset (Fig. 3). At the coding level, both SnpEff and VEP yielded near perfect concordance for SNVs, matching the exact ClinVar nomenclature for over 99.9% of the SNVs (Fig. 5a). However, exact concordance for insertions and deletions, which are often the most clinically relevant variant types, was substantially lower. Notably, concordance for insertions was only 75–80% for both tools. For 16.6% (113/681) of the insertions, the ClinVar syntax appeared to be incorrect due to the failure to right-shift or annotate insertions as duplications. Based on a review of at least 25 variants, (~522/568) was also due to ClinVar right-shifting errors. For 5–10% of these variants, ClinVar did not right-shift correctly. These were deletions at canonical splice sites (e.g., ±2 bases of the intron–exon boundaries) where the right-shifting rule often does not apply; SnpEff and VEP were incorrect for at least 40 and 16 of these variants, respectively. Over half of the SnpEff errors (20/40) were due to the random right-shifting of the variant 11 bases upstream (Additional file 1: Table S8), an error that is now corrected in a newer version of SnpEff (4.2). Altogether, the coding syntax concordance was only 86% for deletions and 88% for insertions, with 8.8% (50/568) of these coding discrepancies causing a change in the protein syntax.

Given the uniformity in SNV syntax, we investigated with interest any discordance in annotations between tools and database (on average 0.5%, 484/85,952). For example, 71.6% (413/582) of SNV discrepancies were off by one base; our inspection revealed that the ClinVar positions were typically correct. Seventy-nine of all discordant SNVs were due to the correct identification of RefSeq-Genomic differences in the ClinVar database. We found eight instances where the tools incorrectly specified the exon boundaries by one or more bases, but could not reconcile these with any discrepancies in the NCBI procured GFF (e.g., rs121434244 and rs786205134 in Additional file 1: Table S9). Finally, for 27 genomic SNVs, ClinVar asserted transcript syntax for insertion and deletion products. These were splice variant products determined uniquely through ClinVar curation, publication, or presumably by the submitters. As example, an SNV at a splice site in the AGA gene (NC_000004.11:g.178354367C > A) results in the skipping of exon 8 and a final syntax of NM_000027.3:c.807_940del134 (Additional file 1: Table S10), demonstrating that there are variants in ClinVar where the predicted consequences are not apparent in the genomic nomenclature as the site of effect is in the RNA.

As with the ground truth test set, we observed greater variation in protein syntax (Table 1). For deletions, duplications, and insertions, this accounted for 16 to 78% of differences (or ~8500 out of 11,945 variants in these variant types). Overall concordance was again high for SNVs (99%; n = 84,982). However, for non-SNV variant types (n = 11,945), between 60 and 70% of annotations by these tools did not match the ClinVar HGVS, and between 5 and 20% of these annotations were completely discordant.

**Table 1:** Exemplar variants demonstrating nomenclature discrepancies

	ClinVar	COSMIC	SnpEff	Vep	VR	Preferred HGVS	Reference ID
Coding HGVS: variant type							
Insertion		c.2339_2340insGGGCTCCCC	c.2331_2339dupGGGCTCCCC	c.2331_2339dupGGGCTCCCC		c.2331_2339dup	COSM12555^*^
Insertion	-	c.2262_2263ins14	-	c.2262_2263insGGCATCTCAGCATC	-	c.2262_2263insGGCATCTCAGCATC	COSM5254274
Deletion	c.1200-1delG		c.1200delG	c.1200delG	c.1201delAinsGA	c.1200-1delG	PTV021, rs63186960
Deletion	c.1895 + 1_1895 + 4delGTGA		c.1895 + 5_1895 + 8delGTGA	c.1895 + 5_1895 + 8delGTGA	c.1895 + 9GTGAC > C	c.1895 + 5_1895 + 8delGTGA	PTV003, rs386834023
Duplication	-	c.422_423insA	c.428dupA	c.428dupA	-	c.428dup	COSM4719972
Indel	-	c.3141_3142GA > TT	c.3141_3142delGAinsTT	c.3141_3142delGAinsTT	-	c.3141_3142delinsTT	COSM4387531
Indel	c.68-5_68-3delinsTT	-	c.68-5_68-3delCTCinsTT	c.68-5_68-3delCTCinsTT	c.68-5_68-3delCTCinsTT	c.68-5_68-3delinsTT	rs397516362
SNV	c.1621A=		c.1621A>G	c.1621A>G	c.1621G=	c.1621G=	PTV099, rs2228006
Protein HGVS: consequence							
Frameshift	p.Arg227Lysfs	-	p.Arg227fs	p.Arg227LysfsTer31	-	All are acceptable	rs80356649
Frameshift	-	p.P1176fs^*^>46	p.Pro1176fs	p.Pro1176AlafsTer117	-	p.Pro1176fs or p.Pro1176AlafsTer117	COSM5196763
Frameshift	-	-	p.Glu238fs	p.Glu238ProfsTer9	p.Phe237_Glu238insPro	p.Glu238fs or p.Glu238ProfsTer9	PTV009
In-frame insertion	-	p.Pro780_Tyr781insGlySerPro	p.Pro780_Tyr781insGlySerPro	p.Gly778_Pro780dup	-	p.Gly778_Pro780dup	COSM12555^1^
Synonymous	p.Arg317=	-	p.Arg317Arg	p.=	p.Arg317=	p.Arg317=	rs111033272
Synonymous	-	p.^*^1143^*^	p.Ter1143Ter	p.=	-	p.Ter1143=	COSM3558732
Stop gained	p.Gln100Ter	-	p.Gln100^*^	p.Gln100Ter	-	All are acceptable	rs119103276
Extention	-	p.^*^1133L	p.Ter1133Leuext^*^?	p.Ter1133LeuextTer22	-	p.Ter1133LeuextTer22	COSM1569676
In-frame insertion	-	-	p.Arg309_Arg310insArgArg	p.Arg310_Arg311dup	p.Arg311_Lys312insArgArg	p.Arg310_Arg311dup	PTV082
In-frame insertion	-	p.T502_H505delTTGH	p.Thr502_His505del	p.Thr502_His505del	-	p.Thr502_His505del	COSM1163654
In-frame deletion	-	-	p.Ala1111_Ala1119del	p.Ala1111_Ala1119del	p.Ala1119_Gly1120insAlaAlaAlaAlaAlaAlaAlaAlaAla	p.Ala1111_Ala1119del	PTV021

^1^ Known in My Cancer Genome as “G778_P780dup”

We isolated for protein syntax differences despite agreement at the coding level. Among these variants (n = 303), most differences in protein syntax were systematic and could be parsed with software: e.g., the reporting of MNVs as substitutions instead of indels (p.AspAla2625GluPro versus p.Asp2625_Ala2626delinsGluPro for rs267606668). Deletions were sometimes annotated distinctly for all three tools (n = 626): for variant NM_001126128.1:c.163delA, ClinVar, SnpEff, and VEP output p.Ile55Terfs, p.Ile55fs, and p.Ile55Ter, respectively. The correct syntax for this variant is p.Ile55Ter.

Areas of inconsistency for protein annotations also included non-coding regions or boundaries between coding and non-coding regions. Though intronic variants are not typically associated with protein annotations, 104 intronic or splice variants had protein changes in ClinVar but no protein annotation by the tools. For example, NM_000090.3:c.951 + 5G > A (rs587779422) is an intronic variant with the ClinVar annotation p.Gly300_Ala317del. References explaining the unusual derivation of protein products from transcript nomenclature, such as in this case, were frequently absent in the ClinVar entry. As another example, ClinVar associates p.Gln2Ter with the 3′ UTR variant NM_005633.3:c.*4C >T, while the tools produced no annotation. Of interest, Mutalyzer asserts that this is a synonymous protein variant (p.(=)).

Overall, we found that the tools were mostly correct. The agreement between SnpEff and VEP on annotations that were together discordant with ClinVar allowed us to identify discrepancies in the ClinVar output. In addition to the right-shifting errors, ClinVar did not provide protein annotations for 353 coding variants. Over 100 variants should have been annotated as duplications instead of insertions. The TTN variant p.Glu3419Asp is incorrectly associated with intronic variant NM_133378.4:c.10303 + 2278G > C, when the correct coding syntax for this protein variant is NM_001267550.2:c.10770G>C. In the process of this analysis, we recognized efforts by ClinVar to resolve these errors by correcting or removing some of the variants described in this paper. This harmonization by ClinVar is encouraging and crucial for ensuring the reliability of this database.

### Comparison with the COSMIC dataset

Clinical cancer care is dependent on identifying relationships between tumor variants and relevant information about their prognostic and therapeutic significance. We investigated the consistency between annotation output by SnpEff and VEP with COSMIC, currently the largest public resource of somatic mutations in human cancer [20] that is also widely used by clinical laboratories. Again, we did not include VR in our assessment because of its limited functionality and long running time. Because COSMIC annotates variants in relation to Ensembl instead of NCBI RefSeq transcript accessions, we built a second, separate database to run VEP and SnpEff according to Ensembl transcript alignments.

We queried a total of 3,076,036 coding COSMIC variants. Following normalization and de-duplication of the COSMIC VCF, there remained a set of 2,215,076 variants, indicating that nearly one-third of the VCFs were duplicate variants. Approximately 142,134 variants were insertions, deletions, or indels, 19% of which required left justification (Additional file 1: Table S2). We compared syntax representations (Fig. 5b; Additional file 6). Both SnpEff and VEP generated annotations for approximately 90% of the COSMIC dataset. Because the cancer field employs the convention of abbreviating amino acids to a single letter while the annotation tools, and HGVS, all use the three-letter convention, we converted the COSMIC annotations to three-letter amino acids to facilitate annotation comparison.

At the coding level, VEP recapitulated the exact syntax as COSMIC for 85.9% of the total variants, compared to 76.8% of variants by SnpEff, with less than 1% of equivalent syntax for both tools (Fig. 5b). However, the majority of the COSMIC dataset are SNVs (95%); for variant types other than SNVs, neither VEP nor SnpEff achieved comparable concordance. Notable differences in annotations include COSMIC’s reporting of all duplications as insertions. Because we did not assert the equivalency of multi-base insertions with duplications due to the involvement of verifying duplicated bases in the reference transcript, this resulted in nearly complete discordance for variants of this type.

For protein variants, SnpEff reproduced the exact protein syntax for 75.8% of COSMIC variants compared to 59.4% by VEP (Fig. 5b). Non HGVS-compliant COSMIC syntax accounted for most discrepancies in exact match (Table 1, Additional file 1: Table S9). For example, a large fraction of VEP and COSMIC differences can be attributed to the correct VEP annotation of frameshifting indels resulting in an immediate termination codon as nonsense variants (e.g., p.Ser6Ter vs p.Ser6fs*1, COSM1476431). COSMIC also incorrectly describes all indels as substitutions (p.Gln256>ArgGlu versus p.Gln256delinsArgGlu, COSM1741200). Over 90% of alternative VEP protein expressions were due to discrepant reporting of synonymous variants as p. = compared to p.Gly35Gly by both COSMIC and SnpEff (Table 1). Nuances in nomenclature revealed distinct expressions of frameshifts for COSMIC, VEP, and SnpEff (Table 1).

Importantly, affecting protein syntax, we found that overall concordance between tools and COSMIC nomenclature for deletions at the coding level was less than 58% (Fig. 5b). As with ClinVar, for the vast majority of discordant annotations, the agreement between SnpEff and VEP syntax suggested that the COSMIC syntax is incorrect. A large proportion of these differences can be attributed to the failure of COSMIC to consistently right justify deletions (Additional file 1: Table S11). To verify the HGVS nomenclature of these variants, we mapped the Ensembl transcript to its approximate corresponding RefSeq accession through its consensus coding sequence, since a number of tools, including Mutalyzer, do not support Ensembl identifiers. A *TP53* variant at position chr17:7578525 (COSM1683507) is annotated in COSMIC as c.404_405insC. Because of a sequence of four Cs at this position, the standardized left-shifted VCF position should be at chr17:7578523 and right-shifted HGVS syntax as c.405_406insC or c.405dupC. In another example, a HER2 insertion variant is described in My Cancer Genome as c.2339_2340ins (with no insertion bases or transcript as reference) and G778_P780dup. The correct coding syntax by both SnpEff and VEP is c.2331_2339dup while the correct protein syntax (output only by VEP) is p.Gly778_Pro780dup. COSMIC incorrectly annotated both coding and protein syntax as c.2332_2340dupGGCTCCCCA and p.Pro780_Tyr781insGlySerPro (Table 1). Based on the agreement of VEP and SnpEff alone, our results suggest that between at least 5 and 10% of COSMIC variant annotations are incorrect. This is concerning given its transition from a research repository to a major clinical resource, although efforts to comply with genomic and HGVS standards are apparently underway.

### Clinical impact of discordant variant annotation

Ultimately, we are concerned about the concordance of positional and syntax expressions because of its impact on clinical interpretation. To illustrate this point, we describe a frameshift variant in the *PROK2* gene, which was differentially classified by two curators in our laboratory—one classified it as likely pathogenic and the other as pathogenic for Kallman syndrome. The difference in classification stemmed from the use of different syntax in constructing the string-based search. The variant was described as “NM_001126128.1:c.297dupT (p.Gly100Trpfs*22)”. Because of alternative transcripts and HGVS representations, this variant could be searched by multiple expressions (Additional file 4: Figure S2a). In one route, searching “PROK2 c.297_298insT” or “PROK2 c.234_235insT” immediately retrieved the relevant literature to classify this variant. However, searching “PROK2 *297_298ins*”, “PROK2 *234_235ins*”, or the correct HGVS syntax “c.297dup” or “c.234dup” did not return any relevant results (Additional file 4: Figures S2b). Searching for “PROK2 G100fsX121”, “PROK2 c.297_298insT” or “PROK2 c.234_235insT” identifies a paper by Abreu et al. [38], which leads to a thread of reports that supports a final variant classification of “pathogenic” (Additional file 4: Figure S2b, c). Because of these multiple variant representations, identifying the relevant information can entail navigating a complex matrix of HGVS expressions and web results.

As another example of the importance of accurate HGVS nomenclature for clinical care, a variant in a patient’s melanoma sample was annotated in our pipeline as “NM_004333.4:c.1799 T > A (p.V600E)”. During visual review we found that the variant was part of a dinucleotide pair, with a combined syntax of c.1799_1800delTGinsAT and protein syntax of p.V600D. Although p.V600D is sensitive to BRAF inhibitors, this variant is not as well-studied and characterized with respect to drug response and efficacy compared to p.V600E. Further, while V600E confers sensitivity to MEK inhibition, the sensitivity of p.V600D to MEK remains unclear.

## Discussion

We have described some of the remaining challenges of moving clinical sequencing into a high-throughput environment. Consistent with findings by McCarthy et al. [17], we find that the transcript collection has a significant impact on the yield of relevant variant annotations. Our examination of automated syntax from HGVS tools and the ClinVar or ground truth datasets reveal that approximately 10% of variants could not be assessed due to discordant transcript accessions or versions. The fact that ClinVar and COSMIC, the largest public repositories of germline and somatic data, respectively, do not share the same collection of transcript accessions reflects the degree of harmonization and the need for a universal store of transcript definitions and genome alignments.

Although annotation resources are updating and improving, any tool or database used for clinical diagnostic purposes should be evaluated with rigorous scrutiny. Our results show that there are resources, including COSMIC and Variation Reporter, that claim to provide HGVS nomenclature when it is clear that they do not always comport with recommended conventions. As these standards are continuously being updated, resources and laboratories employing these annotations must also adapt. We have and continue to share our findings of errors and non-compliance from our analyses with the respective resources, which is critical for improving concordance across laboratories as a community. Our ground truth set has been constructed to test, to the best of our knowledge, the limits of the tools in providing accurate and compliant HGVS annotations. We encourage other laboratories to use this dataset as a quality assurance to evaluate their own in-house annotations with the same rigor.

Importantly, although variant calling is performed almost exclusively on genomic data, variants are still being primarily referenced with respect to the transcript. Recent publications continue to describe variants according to their protein and/or coding syntax [39–41], sometimes even without the transcript identifier [42, 43]. In a survey by the American Society of Molecular Pathologists, 50% of clinical cancer labs report variants exclusively by coding and protein HGVS nomenclature but without accompanying genomic coordinates. The same survey also found that only 77% of clinical cancer laboratories define the transcript in their clinical reports, and at least 70% of labs use as a resource MyCancerGenome.org, which references variants by their popular single-letter amino acid or coding-level convention, again, without transcript or genomic coordinates. As our analyses show, transforming genomic positions to transcript loci is challenging and prone to error; ambiguity in representation is best avoided by always including the variant’s genomic position and assembly version, in addition to the transcript and protein HGVS expressions. For diagnostic applications, the HGVS recommends annotating with respect to the Locus Reference Genomic sequence (LRG) [33], a system designed for clinically relevant variants that is based on un-versioned and stable accession sequences [33, 44]. Although LRG curation is still on-going, we found that, based on the ClinVar XML, approximately 50% of ClinVar entries currently have a LRG assertion.

Despite the precision achieved with generating syntax for SNVs, the positions of insertions and/or deletions remain stubbornly difficult to annotate, regardless of the VCF or HGVS genomic standard. The presence of duplicates in over one-third of the COSMIC VCF highlights the importance of using tools for normalization to reconcile the multiple possible positions representing a single variant. At the level of HGVS, we found that the syntax produced by the tools was far more reliable than the syntax in the ClinVar and COSMIC databases, partly due to consistent right-shifting efforts. However, none of the non-SNV variant types were annotated with near 100% accuracy or compliance with HGVS conventions by either tool or database. Given the meticulous reporting requirements of a clinical genetics lab, this is concerning and suggests that it remains critical to manually review the syntax when reporting non-SNVs.

Our analyses further provide a glimpse into the diverse matrix of possible HGVS representations for a given variant—a challenging concept for attempts to mine and exploit existing resources through string-based search. Within knowledge-bases, internal efforts can be made to standardize HGVS syntax; variants can be transformed into a standard, minimal expression to enable a uniform query across curated databases [45]. While this is useful for a limited set of data, it is impractical for mining beyond internally curated information. The alternative is exhaustive but impractical, requiring the search for every permutation of an HGVS expression for a particular variant. A thoughtful discussion should be made about asserting HGVS guidelines as rules to enforce a strict convergence across laboratories, resources, and literature. Encouragingly, the HGVS has recently restricted the breadth of preferred syntax, though has also added to the challenge of evolving HGVS standards over time.

By design, the HGVS annotation system was never intended for mining large bodies of genomic information, while approximations of syntax are not acceptable because of their impact on clinical care. A means of clinical intervention in oncology is to directly connect clinically actionable variants in patient tumor samples with relevant therapeutic strategies, such as approved drugs or eligibility for clinical trials. In the ACMG guidelines for the classification of germline variants, at least five categories of evidence require interrogating variants from previous reports in reliable databases or the published literature [37]. Already, studies have shown that there remains substantial heterogeneity in the interpretation of genomic variants by clinical laboratories [46]. Imprecise annotation can lead to variant misclassification and misdiagnosis [36] and, in the context of identifying mutant peptides or neoantigens for cancer immunotherapy [47], the design of a potentially ineffective cancer vaccine. The applications of genomics in clinical care will require concerted efforts to converge on standardized reporting mechanisms to enable data sharing and integration across diverse datasets and resources. Including genomic coordinates and reporting on the same genomic reference, according to uniform variant syntax, will be one crucial step towards achieving this aim and the ultimate goal of precision medicine.

## Conclusions

We show that for the HGVS annotation tools evaluated here, the syntax produced for SNVs is generally correct but the syntax for insertions and/or deletions does not meet clinical diagnostic standards and must be manually reviewed for clinical reporting. We further show that for the same variant datasets, there is substantial discordance in syntax between the tools and clinical databases, where the tools are consistently more HGVS compliant. Finally, we provide a ground truth HGVS test set for laboratories and resources to benchmark the accuracy of their annotations. Our results emphasize the urgent need to conform to uniform HGVS syntax, with reporting on the genomic reference, to achieve accurate and precise clinical care.

## Additional files

Additional file 1: Table S1. Ground truth test set. **Table S2.** Variants normalized by dataset. **Table S3.** Ground truth set contents by features. **Table S4.** Ground truth set comparison results. Exact matches between the reference annotation in COSMIC and annotations provided by Snpeff and VEP are marked as “*yes*”, equivalent matches as “*yes_m*” (“*yes modified*”) and not equivalent annotations as “*no*”. **Table S5.** Example nomenclature discrepancies in the ground truth test. **Table S6.** Annotation of dinucleotide substitutions. **Table S7.** Tools run time. **Table S8.** ClinVar variants demonstrating SnpEff right-shifting errors. **Table S9.** Example nomenclature discrepancies in the ClinVar dataset. **Table S10.** Genomic SNVs resulting in deletions at the transcript level. **Table S11.** Example nomenclature discrepancies in the COSMIC dataset. (XLSX 89 kb)
Additional file 2: Input VCF for the ground truth test set. (VCF 8 kb)
Additional file 3: Details related to genome annotation and SnpEff installation. (DOCX 134 kb)
Additional file 4: Supplementary figures. **Figure S1.** Comparison of effect annotation between tools and the HGVS test set. Concordance in effect nomenclature between the ground truth test set and SnpEff and VEP by variant type. **Figure S2.** Impact of HGVS nomenclature on clinical interpretation. **a** Transcripts and nomenclature associated with variant (chr3:g.71821968dupA). **b** PubMed and Google results from search strings. **c** From a single search string to evidence and classification. (PDF 1690 kb)
Additional file 5: ClinVar comparison results. Exact matches between the reference annotation in COSMIC and annotations provided by Snpeff and VEP are noted as “*yes*”, equivalent matches as “*yes_m*” (“*yes modified*”), and not equivalent annotations as “*no*”. (TXT 34402 kb)
Additional file 6: COSMIC comparison results. Exact matches between the reference annotation in COSMIC and annotations provided by Snpeff and VEP are noted as “*yes*”, equivalent matches as “*yes_m*” (“*yes modified*”), and not equivalent annotations as “*no*”. (TXT 385398 kb)

## Abbreviations

COSMIC: Catalogue of Somatic Mutations in Cancer
HGVS: Human Genome Variation Society
MNV: Multinucleotide variant
SNV: Single nucleotide variant
VCF: Variant Call Format
VEP: Variation Effect Predictor
VR: Variation Reporter
