# Supplementary material for: A variant by any name: quantifying annotation discordance across tools and clinical databases
Source: Genome Med. 2017 Jan 26;9:7. doi: 10.1186/s13073-016-0396-7 (PMC5267466; doi:10.1186/s13073-016-0396-7)
Supplement: Additional file 3: — Details related to genome annotation and SnpEff installation. (DOCX 134 kb) [file 13073_2016_396_MOESM3_ESM.docx]

**Additional File 3: Genome annotation and SnpEff installation**

Our genome annotations are based on the latest version of the NCBI eukaryotic genome annotation pipeline [1] based on GRCh37 (v105). We built a SnpEff prediction database using the annotation release 105 GFF3 file ([ref_GRCh37.p13_top_level.gff3.gz](ftp://ftp.ncbi.nih.gov/genomes/Homo_sapiens/ARCHIVE/ANNOTATION_RELEASE.105/GFF/ref_GRCh37.p13_top_level.gff3.gz)) obtained from NCBI with associated transcripts ([rna.fa.gz](ftp://ftp.ncbi.nih.gov/genomes/Homo_sapiens/ARCHIVE/ANNOTATION_RELEASE.105/RNA/rna.fa.gz)) and protein sequences from that same release ([protein.fa.gz](ftp://ftp.ncbi.nih.gov/genomes/Homo_sapiens/ARCHIVE/ANNOTATION_RELEASE.105/protein/protein.fa.gz)). In order to conform with expectations of the SnpEff software, a number of modifications had to be made to the FASTA sequences from NCBI. The GFF data was also modified to convert the sequence names from RefSeq accessions ("NC_000022.10”) to assembly sequence names ("22") for consistency with our analysis pipelines and our genome FASTA file. That was the only modification made to the GFF.

Example transcript data row from NCBI GFF after modification:

1    BestRefSeq      mRNA    69091   70008   .       +       .       ID=rna5;Name=NM_001005484.1;Parent=gene6;Dbxref=GeneID:79501,Genbank:NM_001005484.1,HGNC:14825,HPRD:14974;gbkey=mRNA;gene=OR4F5;product=olfactory receptor%2C family 4%2C subfamily F%2C member 5;transcript_id=NM_001005484.1

The NCBI FASTA sequence data were modified to use GFF object identifiers instead of NCBI identifiers since SnpEff keys on GFF ID attribute for connecting transcripts and protein identifiers present in the GFF file to the sequence data in the FASTA files. Shown below is an example FASTA sequence identifier line before and after modification:

Transcript FASTA original:
>gi|53828739|ref|NM_001005484.1| Homo sapiens olfactory receptor, family 4, subfamily F, member 5 (OR4F5), mRNA

Transcript FASTA modified:
>rna5 gi|53828739|ref|NM_001005484.1| Homo sapiens olfactory receptor, family 4, subfamily F, member 5 (OR4F5), mRNA

Protein FASTA modified: 
>rna5 gi|53828740|ref|NP_001005484.1| olfactory receptor 4F5 [Homo sapiens]

SnpEff uses the transcript identifiers when looking up both the transcript's sequence and the corresponding protein sequence. So, in order for SnpEff to be able to process the NCBI GFF data, the identifier lines for both transcript and protein FASTA sequence files were modified to use the GFF object identifier for the transcript.

Our raw SnpEff results contain GFF object identifiers which we subsequently map to RefSeq accessions as part of our post-processing after running SnpEff.

In our initial attempt to use SnpEff v4.2 at the time of manuscript preparation, we encountered a bug related to HGVS annotation [2] leading us to decide to proceed with SnpEff v4.1L. We have also been testing SnpEff v4.3, however additional bugs were encountered [3].

1. NCBI. The NCBI Eukaryotic Genome Annotation Pipeline [Internet]. [cited 2016 Nov 4]. Available from: https://www.ncbi.nlm.nih.gov/genome/annotation_euk/process/

2. HGVS interval error on structural variants near the ends of chromosomes · Issue #128 · pcingola/SnpEff. Available from: https://github.com/pcingola/SnpEff/issues/128

3. SnpEff 4.3 Macaca fascicularis, Interval error: end before start #154 [Internet]. [cited 2016 Nov 4]. Available from: https://github.com/pcingola/SnpEff/issues/154
