# Supplementary material for: A variant by any name: quantifying annotation discordance across tools and clinical databases
Source: Genome Med. 2017 Jan 26;9:7. doi: 10.1186/s13073-016-0396-7 (PMC5267466; doi:10.1186/s13073-016-0396-7)

S1

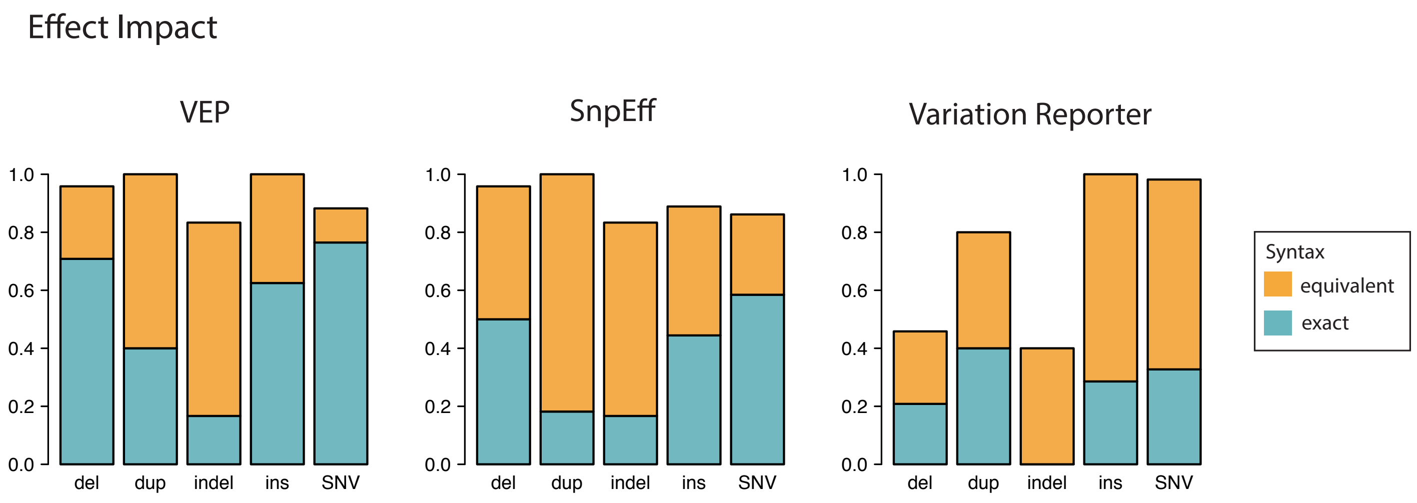
Effect Impact
VEP
SnpEff
Variation Reporter
1.0
0.8
0.6
0.4
0.2
0.0
del
dup
indel
ins
SNV
Syntax
equivalent
exact

S2

a. Possible transcripts and nomenclature

Transcripts	Coding HGVS	Protein HGVS
NM_001126128.1	c.297dupT	p.Gly100Trpfs*22
NM_021935.3	c.234dupT	p.Gly79Trpfs*22

b. HGVS syntax search matrix

**Search**	**Result - PubMed**	**Result - Google**
PROK2 297dup	(nothing)	LOVD entry from ESP
PROK2 Gly100Trpfs OR Gly100fs OR G100Wfs OR G100fs	(nothing)	LOVD entry from ESP; Dissertation in Portuguese
PROK2 234dup	(nothing)	(nothing)
PROK2 Gly79Trpfs OR Gly79fs OR G79Wfs OR G79fs	(nothing)	Link to company primer assay webpage
**PROK2 G100fsX121**	**Abreu et al., 2008**	**Abreu et al., 2008; other review papers**

c. Spider-webbing to evidence and classification

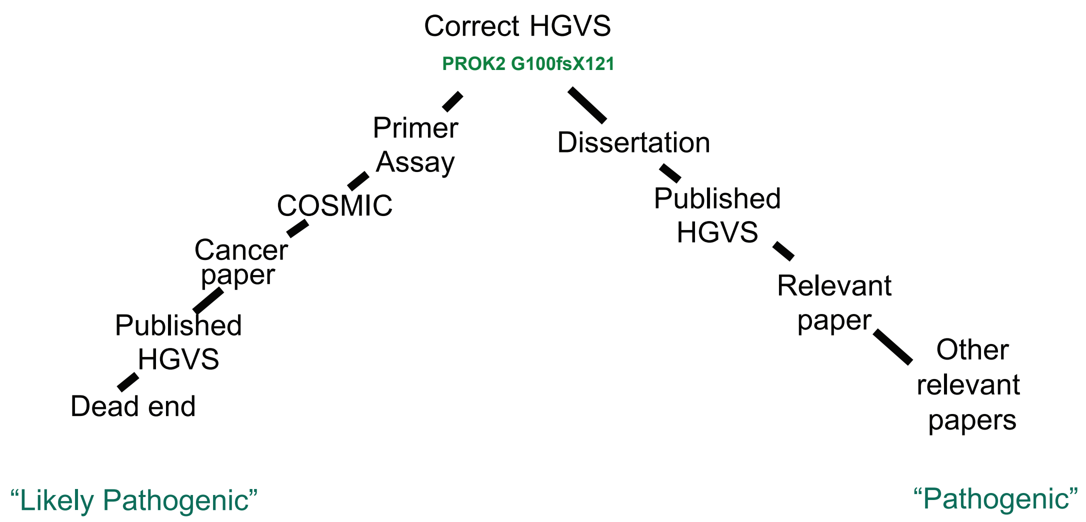

Supplement: Additional file 4: — Supplementary figures. Figure S1. Comparison of effect annotation between tools and the HGVS test set. Concordance in effect nomenclature between the ground truth test set and SnpEff and VEP by variant type. Figure S2. Impact of HGVS nomenclature on clinical interpretation. a Transcripts and nomenclature associated with variant (chr3:g.71821968dupA). b PubMed and Google results from search strings. c From a single search string to evidence and classification. (PDF 1690 kb) [file 13073_2016_396_MOESM4_ESM.pdf]
